# Supplementary material for: Worldwide inequality in access to full text scientific articles: the example of ophthalmology
Source: PeerJ. 2019 Oct 30;7:e7850. doi: 10.7717/peerj.7850 (PMC6825414; doi:10.7717/peerj.7850)
Supplement: Supplemental Information 2 [file peerj-07-7850-s002.docx]

**Supplemental_information 2: Protocol sent to participants in Hinari eligible countries**

Dear Colleague,

Please find enclosed the research protocol for the study “Worldwide accessibility to scientific articles: the example of ophthalmology” to which you have agreed to participate.

For the interest of the study and to avoid bias in methodology please, respect each instruction. If you have any question or problem please contact Christophe Boudry (christophe.boudry@chartes.psl.eu)

As your country is eligible for HINARI program^[[1]](#footnote-1)^, please contact the Director of your institution and/or the administration staff of your library to know if your institution has specific access to Hinari resources. If it is the case, your institution will give you a login and password to access these resources. You will have to login using the “Research4life” site web: <http://login.research4life.org/tacgw/login.cshtml>. The search of the articles must be done once connected to this access.

If you can’t have access to HINARI resources, please use your institutional computer and institutional internet network to search articles (nomadic access outside institutions must be avoided except if there is no other solutions to access to internet network).

We have randomly selected 200 articles in the field of ophthalmology published in 2017 or 2018 in English, which were extracted from PubMed using the keyword (MeSH term) “eye diseases”. Out of these 200 articles, 85 are open access and 115 are behind paywalls. Thus, the objective of is to assess the accessibility of the PDF of those 115 articles for all the participants of the study. The references of these 115 articles are in the files “results.docx” or “results.xlsx”, at your convenience depending on which software (Word or Excel) you prefer to use.

1. For researching the articles: enter the address (from your web browser): <https://www.ncbi.nlm.nih.gov/pubmed/> to access PubMed website. Copy the Digital Object Identifier (DOI)^[[2]](#footnote-2)^ situated in the second column (in the files “results.docx” **or** “results.xls). Please do not modify the ID number in the first column which will be useful for the data analysis.


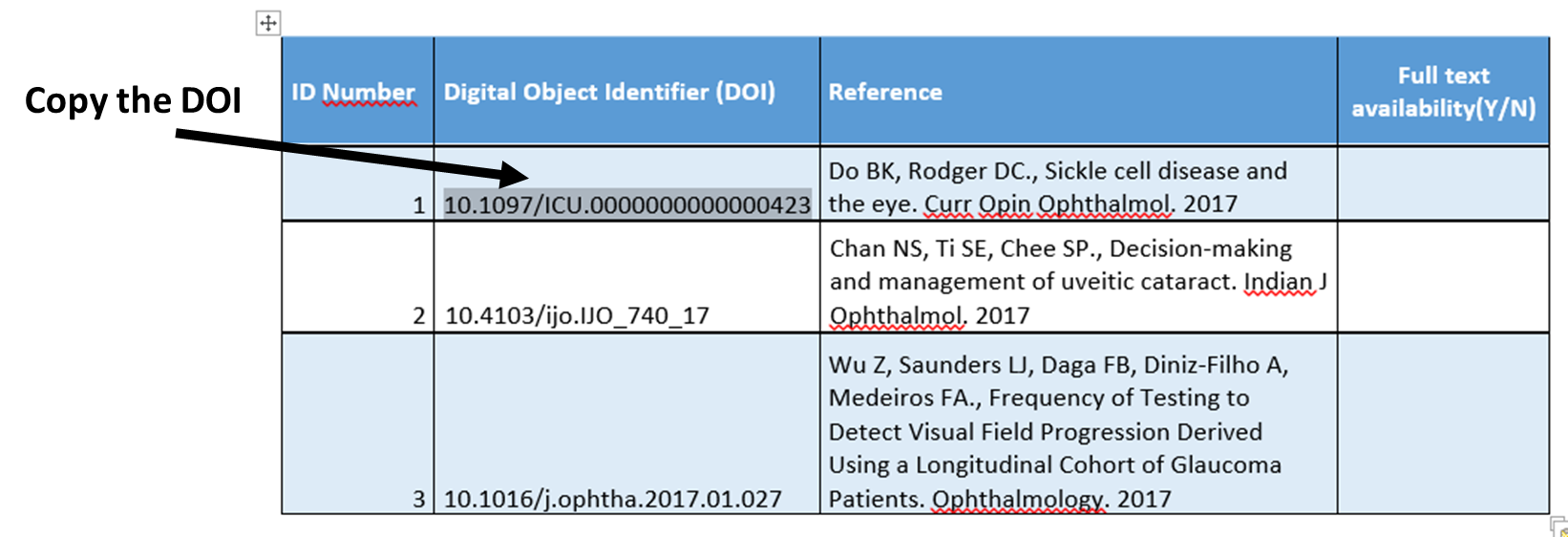


2. Paste it on the PubMed search box (see the figure below). Then, click on the “Search” button.


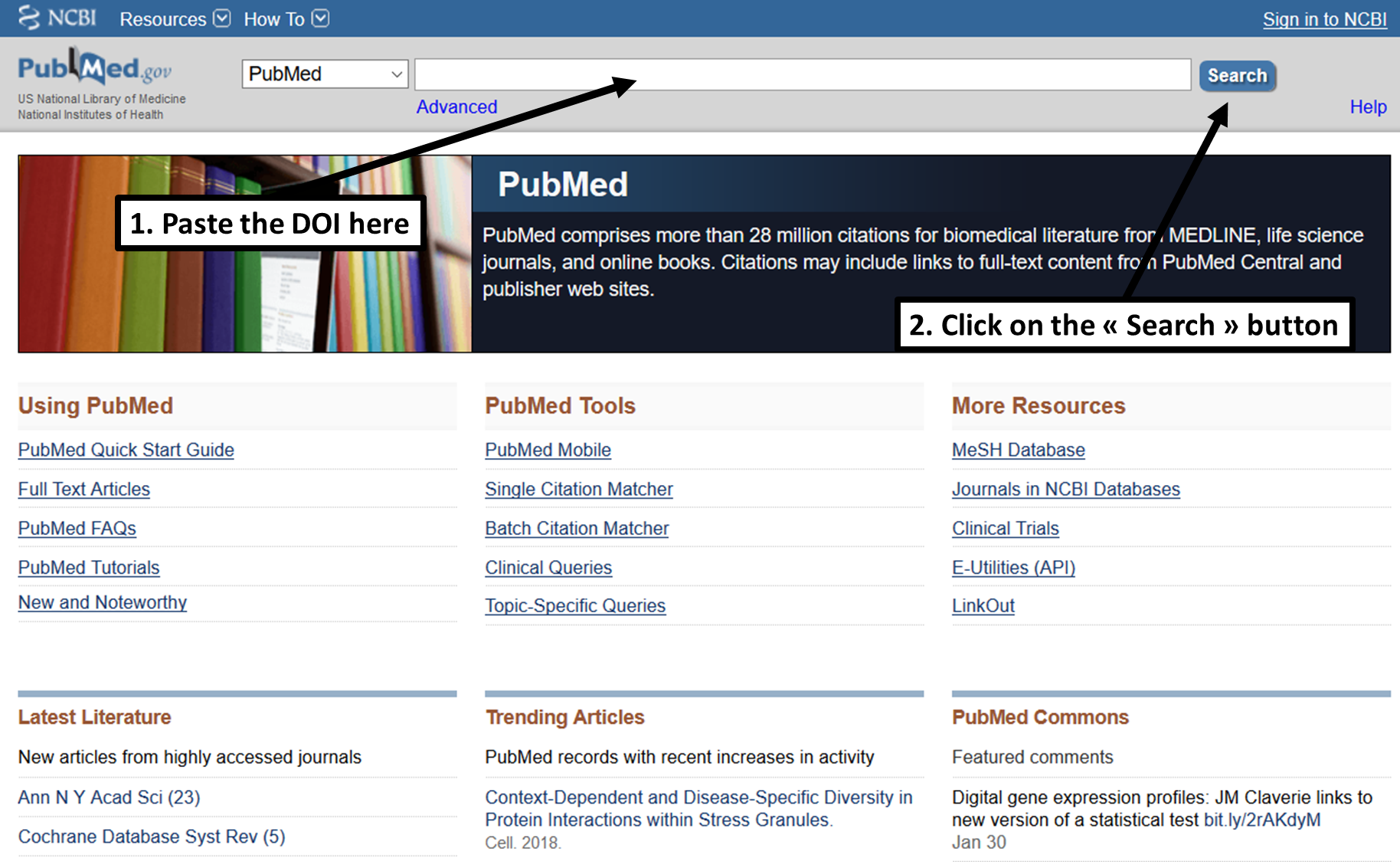


This action brings you to the PubMed results page.

3. Click on the editor icon to access the article on the editor’s website (in the upper right corner called “Full text links”), as shown in the example below (Wolters Kluwer in this example). Please ignore the PMC icon if it is present.


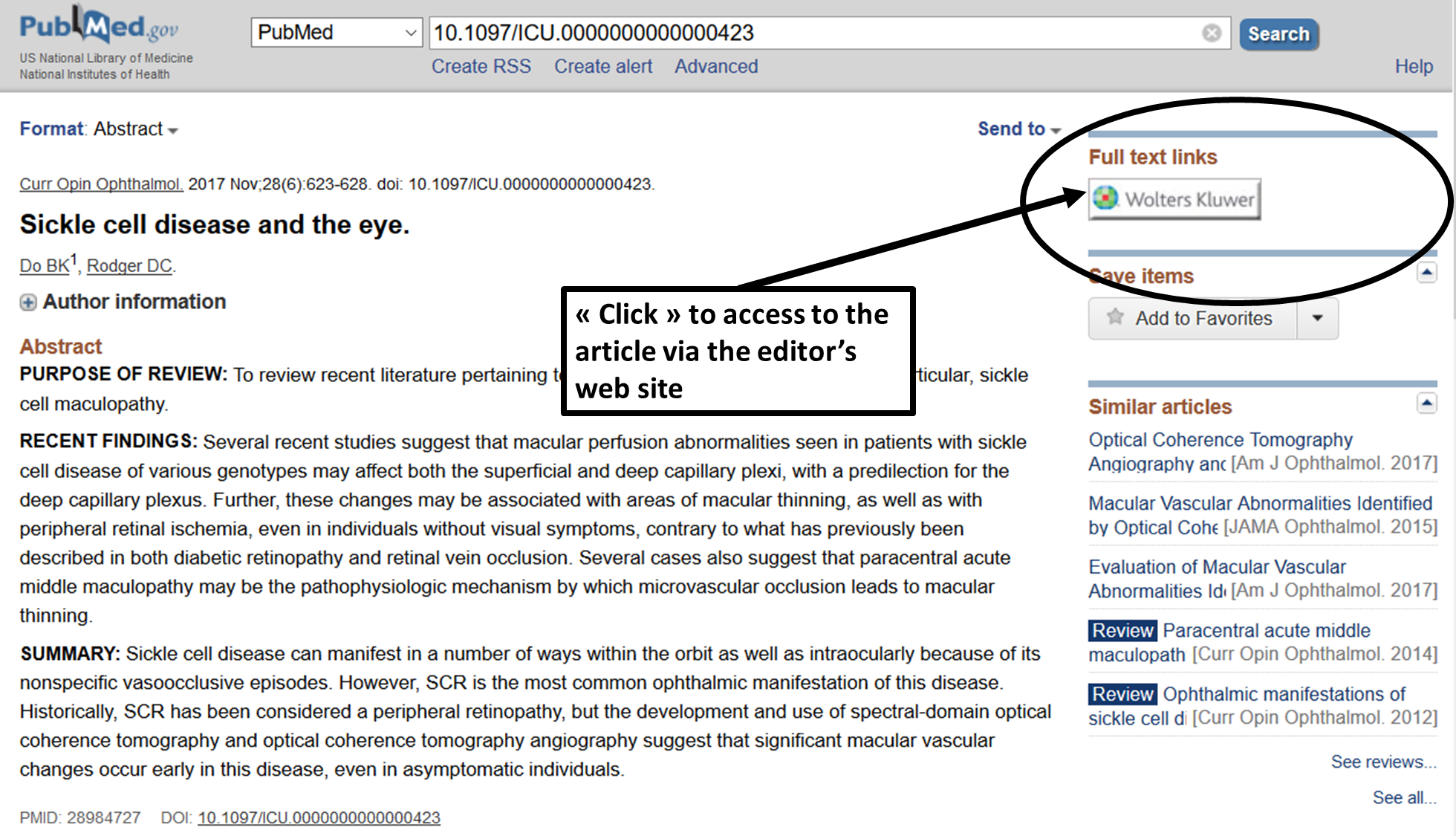


Depending on your institution, you may have an additional specific icon alone or with the icon of the publisher (see below the example of the University of Laval, Canada):


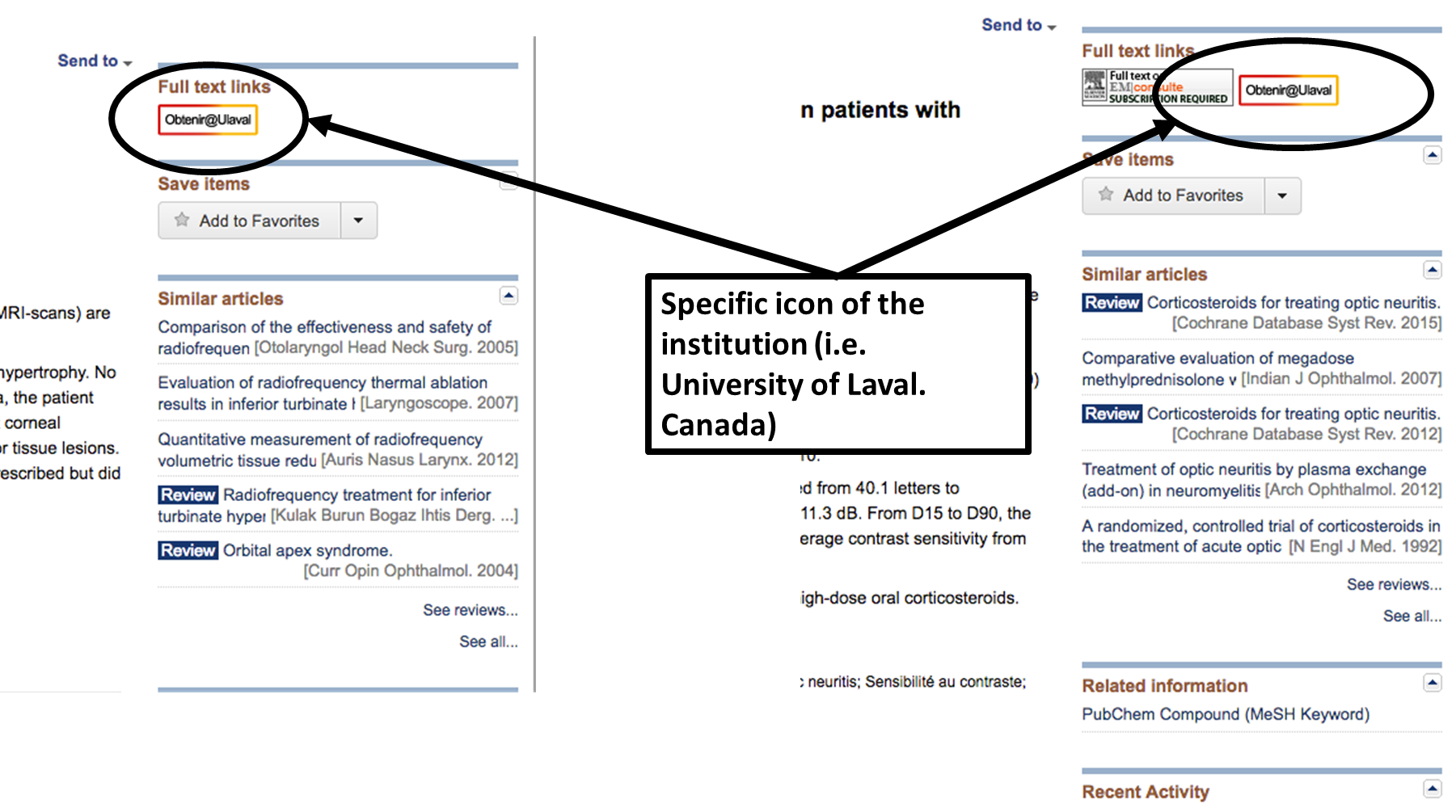


4. Then please click on this specific icon to access the article.

5. Once on the article’s web page, find the link to the full text of the article and try to access the PDF file. Please note that you must verify whether the PDF is downloadable or not on your computer to be sure that the access is possible or not (this ensures that you can pass through the paywall and that you really have access to the full text).

6. Please record in the file “results.docx” or “results.xlsx” (at you convenience) if you can access the PDF (Y) or not (N) in the column “Access to full text (Y/N)”, as shown in the example below.

| **Digital Object Identifier (DOI)** | **Reference** | **Access to full Text (Y/N)** |
| --- | --- | --- |
|  |  |  |
| 10.1097/ICU.0000000000000423 | Do BK, Rodger DC., Sickle cell disease and the eye. Curr Opin Ophthalmol. 2017 | Y |
| 10.4103/ijo.IJO_740_17 | Chan NS, Ti SE, Chee SP., Decision-making and management of uveitic cataract. Indian J Ophthalmol. 2017 | N |
| 10.1016/j.ophtha.2017.01.027 | Wu Z, Saunders LJ, Daga FB, Diniz-Filho A, Medeiros FA., Frequency of Testing to Detect Visual Field Progression Derived Using a Longitudinal Cohort of Glaucoma Patients. Ophthalmology. 2017 | Y |

If you have any questions, feel free to contact us.

When you have verified the search for all articles and recorded all results, please send us back the file “results.docx” or “results.xlsx” (at your convenience), and this file after answering the questions below at these email addresses christophe.boudry@chartes.psl.eu and Frederic.MOURIAUX@chu-rennes.fr.

Thank you so much for your participation and confidence.

C. Boudry and F. Mouriaux

1. Hinari Programme set up by WHO together with major publishers, enables low- and middle- income countries to gain access to one of the world's largest collections of biomedical and health literature. Up to 14,000 journals (in 45 different languages), up to 56,000 e-books, up to 120 other information resources are now available to health institutions in more than 115 countries, areas and territories benefiting many thousands of health workers and researchers, and in turn, contributing to improve world health. [http://www.who.int/hinari/en/] [↑](#footnote-ref-1)
2. A DOI is a persistent and reliable unique identifier to manage access to digital resources, especially scientific articles, allowing to access articles via this identifier [↑](#footnote-ref-2)
