# Supplementary material for: Worldwide inequality in access to full text scientific articles: the example of ophthalmology
Source: PeerJ. 2019 Oct 30;7:e7850. doi: 10.7717/peerj.7850 (PMC6825414; doi:10.7717/peerj.7850)
Supplement: Supplemental Information 3 [file peerj-07-7850-s003.docx]

**Supplemental_information 3: List of “paywalled articles” to be searched for by all participants**

| **ID Number (please do not modify)** | **Digital Object Identifier (DOI)** | **Reference** | **Access to Full text (Y/N)** |
| --- | --- | --- | --- |
| 1 | 10.1016/j.neulet.2016.12.057 | Azuchi Y, Kimura A, Guo X, Akiyama G, Noro T, Harada C, Nishigaki A, Namekata K, Harada T., Valproic acid and ASK1 deficiency ameliorate optic neuritis and neurodegeneration in an animal model of multiple sclerosis. Neurosci Lett. 2017 |  |
| 2 | 10.1111/cge.12895 | Kletke S, Batmanabane V, Dai T, Vincent A, Li S, Gordon KA, Papsin BC, Cushing SL, Héon E., The combination of vestibular impairment and congenital sensorineural hearing loss predisposes patients to ocular anomalies, including Usher syndrome. Clin Genet. 2017 |  |
| 3 | 10.1097/DSS.0000000000000787 | Martínez-Palmer A, Calsina-Prat M, Ormaechea N, Toll A., Reconstruction of Combined Upper and Lower Eyelid Defects in a Patient With Lentigo Maligna. Dermatol Surg. 2017 |  |
| 4 | 10.1093/brain/awx236 | Niturad CE, Lev D, Kalscheuer VM, Charzewska A, Schubert J, Lerman-Sagie T, Kroes HY, Oegema R, Traverso M, Specchio N, Lassota M, Chelly J, Bennett-Back O, Carmi N, Koffler-Brill T, Iacomino M, Trivisano M, Capovilla G, Striano P, Nawara M, Rzonca S, Fischer U, et al., Rare GABRA3 variants are associated with epileptic seizures, encephalopathy and dysmorphic features. Brain. 2017 |  |
| 5 | 10.1080/13816810.2016.1188124 | Tyagi P, Juma Z, Reddy AR., Retinal features in Mulvihill-Smith syndrome. Ophthalmic Genet. 2017 |  |
| 6 | 10.1097/WNO.0000000000000455 | Mendes Marques NBPS, Barros SR, Miranda AF, Nobre Cardoso J, Parreira S, Fonseca T, Donaire NM, Campos N., Horizontal Gaze Palsy and Progressive Scoliosis With ROBO 3 Mutations in Patients From Cape Verde. J Neuroophthalmol. 2017 |  |
| 7 | 10.1080/09286586.2017.1313992 | Oye J, Mactaggart I, Polack S, Schmidt E, Tamo V, Okwen M, Kuper H., Prevalence and Causes of Visual Impairment in Fundong District, North West Cameroon: Results of a Population-Based Survey. Ophthalmic Epidemiol. 2017 |  |
| 8 | 10.1016/j.clae.2017.01.003 | Gifford K, Gifford P, Hendicott PL, Schmid KL., Near binocular visual function in young adult orthokeratology versus soft contact lens wearers. Cont Lens Anterior Eye. 2017 |  |
| 9 | 10.2217/bmm-2017-0218 | Wostyn P, De Deyn PP., Intracranial pressure-induced optic nerve sheath response as a predictive biomarker for optic disc edema in astronauts. Biomark Med. 2017 |  |
| 10 | 10.1097/WNO.0000000000000555 | Morrow MJ, Ko MW., Should Oral Corticosteroids Be Used to Treat Demyelinating Optic Neuritis? J Neuroophthalmol. 2017 |  |
| 11 | 10.1080/09273972.2017.1349816 | Morad Y, Pras E, Nemet A., Superior and Lateral Rectus Myopexy for Acquired Adult Distance Esotropia: A "One Size Fits All" Surgery. Strabismus. 2017 |  |
| 12 | 10.1097/IOP.0000000000000731 | Repp DJ, Rubinstein TJ, Herreid PA, Sires BS., Biomechanics of the Levator Aponeurosis. Ophthal Plast Reconstr Surg. 2017 |  |
| 13 | 10.1007/s00417-017-3817-4 | De Gregorio A, Pedrotti E, Stevan G, Montali M, Morselli S., Safety and efficacy of multiple cyclocoagulation of ciliary bodies by high-intensity focused ultrasound in patients with glaucoma. Graefes Arch Clin Exp Ophthalmol. 2017 |  |
| 14 | 10.1111/aos.13445 | Ten Berge JCEM, van Dijk EHC, Schreurs MWJ, Vermeer J, Boon CJF, Rothova A., Antiretinal antibodies in central serous chorioretinopathy: prevalence and clinical implications. Acta Ophthalmol. 2018 |  |
| 15 | 10.1007/s10072-016-2785-5 | Kang H, Kim SK, Park KJ, Choi NC, Kwon OY, Lim BH., Hemianopic line bisection error in a patient with Alzheimer's disease. Neurol Sci. 2017 |  |
| 16 | 10.1212/WNL.0000000000004312 | Hennes EM, Baumann M, Schanda K, Anlar B, Bajer-Kornek B, Blaschek A, Brantner-Inthaler S, Diepold K, Eisenkölbl A, Gotwald T, Kuchukhidze G, Gruber-Sedlmayr U, Häusler M, Höftberger R, Karenfort M, Klein A, Koch J, Kraus V, Lechner C, Leiz S, Leypoldt F, Mader S, et al., Prognostic relevance of MOG antibodies in children with an acquired demyelinating syndrome. Neurology. 2017 |  |
| 17 | 10.1016/j.oftal.2016.08.006 | Pareja-Ríos A, Bonaque-González S, Serrano-García M, Cabrera-López F, Abreu-Reyes P, Marrero-Saavedra MD., Tele-ophthalmology for diabetic retinopathy screening: 8 years of experience. Arch Soc Esp Oftalmol. 2017 |  |
| 18 | 10.1007/s00417-016-3530-8 | Mikhail M, El-Rayes EN, Kojima K, Ajlan R, Rezende F., Catheter-guided suprachoroidal buckling of rhegmatogenous retinal detachments secondary to peripheral retinal breaks. Graefes Arch Clin Exp Ophthalmol. 2017 |  |
| 19 | 10.1007/s00417-017-3616-y | Shah MA, Agrawal R, Teoh R, Shah SM, Patel K, Gupta S, Gosai S., Pediatric ocular trauma score as a prognostic tool in the management of pediatric traumatic cataracts. Graefes Arch Clin Exp Ophthalmol. 2017 |  |
| 20 | 10.1016/j.jfo.2016.09.014 | Zaarour K, Slim E, Antoun J, Waked N., Thick keratoconic cornea associated with posterior polymorphous corneal dystrophy. J Fr Ophtalmol. 2017 |  |
| 21 | 10.1097/IAE.0000000000001311 | Shifera AS, Pennesi ME, Yang P, Lin P., ULTRA-WIDE-FIELD FUNDUS AUTOFLUORESCENCE FINDINGS IN PATIENTS WITH ACUTE ZONAL OCCULT OUTER RETINOPATHY. Retina. 2017 |  |
| 22 | 10.1080/13816810.2017.1354384 | Bryant L, Lozynska O, Han G, Morgan JIW, Gai X, Maguire AM, Aleman T, Bennett J., On variants and disease-causing mutations: Case studies of a SEMA4A variant identified in inherited blindness. Ophthalmic Genet. 2018 |  |
| 23 | 10.1097/IAE.0000000000001391 | Cheung CMG, Yanagi Y, Mohla A, Lee SY, Mathur R, Chan CM, Yeo I, Wong TY., CHARACTERIZATION AND DIFFERENTIATION OF POLYPOIDAL CHOROIDAL VASCULOPATHY USING SWEPT SOURCE OPTICAL COHERENCE TOMOGRAPHY ANGIOGRAPHY. Retina. 2017 |  |
| 24 | 10.1016/j.jcjo.2016.08.003 | Lassalle S, Maschi C, Caujolle JP, Giordanengo V, Hofman P., Inverted conjunctival papilloma: a certainly underestimated high-risk lesion for carcinomatous transformation-a case report. Can J Ophthalmol. 2017 |  |
| 25 | 10.2217/epi-2017-0060 | Piras IS, Mills G, Llaci L, Naymik M, Ramsey K, Belnap N, Balak CD, Jepsen WM, Szelinger S, Siniard AL, Lewis CR, LaFleur M, Richholt RF, De Both MD, Avela K, Rangasamy S, Craig DW, Narayanan V, Järvelä I, Huentelman MJ, Schrauwen I., Exploring genome-wide DNA methylation patterns in Aicardi syndrome. Epigenomics. 2017 |  |
| 26 | 10.1097/IJG.0000000000000551 | Perez CI, Mellado F, Jones A, Colvin R., Trabeculectomy Combined With Collagen Matrix Implant (Ologen). J Glaucoma. 2017 |  |
| 27 | 10.1016/j.jcjo.2016.07.015 | De Oliveira PR, Berger AR, Chow DR., Optical coherence tomography angiography in chorioretinal disorders. Can J Ophthalmol. 2017 |  |
| 28 | 10.1016/j.jneuroim.2017.07.006 | Yoshikura N, Kimura A, Hayashi Y, Inuzuka T., Anti-C1q autoantibodies in patients with neuromyelitis optica spectrum disorders. J Neuroimmunol. 2017 |  |
| 29 | 10.1097/SCS.0000000000003431 | Na J, Choi SY, Baek S, Lee H., Hemorrhage and Infarction of the Conjunctiva and Orbit in Essential Thrombocythemia. J Craniofac Surg. 2017 |  |
| 30 | 10.1097/ICB.0000000000000374 | Despréaux R, Mrejen S, Quentel G, Cohen SY., EN FACE OPTICAL COHERENCE TOMOGRAPHY (OCT) AND OCT ANGIOGRAPHY FINDINGS IN RETINAL ASTROCYTIC HAMARTOMAS. Retin Cases Brief Rep. 2017 |  |
| 31 | 10.1136/bjophthalmol-2016-309996 | Dagi LR, MacKinnon S, Zurakowski D, Prabhu SP., Rectus muscle excyclorotation and V-pattern strabismus: a quantitative appraisal of clinical relevance in syndromic craniosynostosis. Br J Ophthalmol. 2017 |  |
| 32 | 10.1097/IAE.0000000000001351 | Medina CA, Ortiz AG, Relhan N, Smiddy WE, Townsend JH, Flynn HW Jr., MACULAR HOLE AFTER PARS PLANA VITRECTOMY FOR RHEGMATOGENOUS RETINAL DETACHMENT. Retina. 2017 |  |
| 33 | 10.1080/09286586.2016.1257027 | Malkin AG, Goldstein JE, Massof RW., Multivariable Regression Model of the EuroQol 5-Dimension Questionnaire in Patients Seeking Outpatient Low Vision Rehabilitation. Ophthalmic Epidemiol. 2017 |  |
| 34 | 10.1007/s00018-017-2643-5 | Kudo A., Introductory review: periostin-gene and protein structure. Cell Mol Life Sci. 2017 |  |
| 35 | 10.1080/02713683.2017.1344712 | Wang T, Zhu L, Peng Y, Shen N, Yu Y, Yao M, Zhu J., Photochemical Cross-Linking for Penetrating Corneal Wound Closure in Enucleated Porcine Eyes. Curr Eye Res. 2017 |  |
| 36 | 10.1093/brain/awx219 | Gerber S, Charif M, Chevrollier A, Chaumette T, Angebault C, Kane MS, Paris A, Alban J, Quiles M, Delettre C, Bonneau D, Procaccio V, Amati-Bonneau P, Reynier P, Leruez S, Calmon R, Boddaert N, Funalot B, Rio M, Bouccara D, Meunier I, Sesaki H, et al., Mutations in DNM1L, as in OPA1, result indominant optic atrophy despite opposite effectson mitochondrial fusion and fission. Brain. 2017 |  |
| 37 | 10.1097/IAE.0000000000001683 | Cheng YC, Shen JH, Chao AN, Chen KJ., Later Development of Posterior Staphyloma in Choroidal Osteoma With Choroidal Neovascularization. Retina. 2017 |  |
| 38 | 10.2174/1567202614666161104115440 | Shimazawa M, Inoue Y, Masuda T, Onodera R, Tahara K, Shimizu Y, Mibe Y, Tsuruma K, Takeuchi H, Hara H., Topical Diclofenac-Loaded Liposomes Ameliorate Laser-Induced Choroidal Neovascularization in Mice and Non-Human Primates. Curr Neurovasc Res. 2017 |  |
| 39 | 10.1007/s00405-016-4308-7 | Ozsutcu M, Balci O, Tanriverdi C, Demirci G., Efficacy of adjunctive mitomycin C in transcanalicular diode laser dacryocystorhinostomy. Eur Arch Otorhinolaryngol. 2017 |  |
| 40 | 10.1136/bjophthalmol-2016-309775 | Aixinjueluo W, Usui T, Miyai T, Toyono T, Sakisaka T, Yamagami S., Accelerated transepithelial corneal cross-linking for progressive keratoconus: a prospective study of 12 months. Br J Ophthalmol. 2017 |  |
| 41 | 10.1016/j.ijporl.2016.11.027 | Yin T, van der Meer G., Neonatal airway obstruction in bilateral congenital dacryocystocoele: Case report and review of the literature. Int J Pediatr Otorhinolaryngol. 2017 |  |
| 42 | 10.1007/s00417-017-3860-1 | Chan JCW, Choy BNK, Chan OCC, Li KKW., Early intraocular pressure change after peripheral iridotomy with ultralow fluence pattern scanning laser and Nd:YAG laser in primary angle-closure suspect: Kowloon East Pattern Scanning Laser Study Report No. 3. Graefes Arch Clin Exp Ophthalmol. 2018 |  |
| 43 | 10.1136/bjophthalmol-2016-309245 | Sharma S, Ang M, Najjar RP, Sng C, Cheung CY, Rukmini AV, Schmetterer L, Milea D., Optical coherence tomography angiography in acute non-arteritic anterior ischaemic optic neuropathy. Br J Ophthalmol. 2017 |  |
| 44 | 10.1080/01676830.2017.1279664 | Medel R, Balaguer Solé Ò, Vasquez LM., Inferior tarsal muscle-conjunctivectomy for reverse ptosis repair: A novel technique. Orbit. 2017 |  |
| 45 | 10.1038/eye.2017.25 | Betts T, Ahmed S, Maguire S, Watts P., Characteristics of non-vitreoretinal ocular injury in child maltreatment: a systematic review. Eye (Lond). 2017 |  |
| 46 | 10.1007/s10792-016-0261-0 | Altunel O, Duru N, Goktas A, Ozkose A, Goktas E, Atas M., Evaluation of foveal photoreceptor layer in eyes with macular edema associated with branch retinal vein occlusion after ozurdex treatment. Int Ophthalmol. 2017 |  |
| 47 | 10.1159/000478705 | Motohashi R, Noma H, Yasuda K, Kotake O, Goto H, Shimura M., Dynamics of Inflammatory Factors in Aqueous Humor during Ranibizumab or Aflibercept Treatment for Age-Related Macular Degeneration. Ophthalmic Res. 2017 |  |
| 48 | 10.1016/j.jns.2017.01.033 | Kang S, Shaikh AG., Acquired pendular nystagmus. J Neurol Sci. 2017 |  |
| 49 | 10.1097/WNO.0000000000000503 | Sadaka A, Schockman SL, Golnik KC., Evaluation of Horner Syndrome in the MRI Era. J Neuroophthalmol. 2017 |  |
| 50 | 10.1136/bjophthalmol-2016-310105 | Jirawison C, Liu Y, Surasit K, Maningding E, Kamphaengkham S, Ausayakhun S, Heiden D, Margolis TP, Gonzales JA, Acharya NR, Keenan JD., Fundus findings in a series of patients with extrapulmonary tuberculosis in Thailand. Br J Ophthalmol. 2017 |  |
| 51 | 10.1016/j.neuroimage.2016.12.053 | Pelland M, Orban P, Dansereau C, Lepore F, Bellec P, Collignon O., State-dependent modulation of functional connectivity in early blind individuals. Neuroimage. 2017 |  |
| 52 | 10.1097/ICO.0000000000001130 | Zhang X, Bitar M, Davis RM., Spontaneous Canalicular Plug Erosion After Punctal Plug Placement. Cornea. 2017 |  |
| 53 | 10.3928/23258160-20171030-04 | Stephens JD, Adam MK, Todorich B, Faia LJ, Garg S, Dunn JP, Mehta S., Optical Coherence Tomography Findings in Endogenous Fungal Chorioretinitis, Retinitis, and Endophthalmitis. Ophthalmic Surg Lasers Imaging Retina. 2017 |  |
| 54 | 10.1097/ICL.0000000000000297 | Hashemi H, Rastad H, Emamian MH, Fotouhi A., Floppy Eyelid Syndrome and Its Determinants in Iranian Adults: A Population-Based Study. Eye Contact Lens. 2017 |  |
| 55 | 10.1177/2165079916686592 | Lurati AR., Identifying Personal Risk Factors for Falls in the Workplace. Workplace Health Saf. 2017 |  |
| 56 | 10.1016/j.ad.2016.07.006 | Miura T, Ohtsuka M, Yamamoto T., Sweet's syndrome-like eruption in association with the exacerbation of Behçet's disease after the Great East Japan Earthquake. Actas Dermosifiliogr. 2017 |  |
| 57 | 10.1001/jamaophthalmol.2017.1009 | Borkar DS, Homayounfar G, Tham VM, Ray KJ, Vinoya AC, Uchida A, Acharya NR., Association Between Thyroid Disease and Uveitis: Results From the Pacific Ocular Inflammation Study. JAMA Ophthalmol. 2017 |  |
| 58 | 10.1111/opo.12332 | Li SM, Kang MT, Wu SS, Meng B, Sun YY, Wei SF, Liu L, Peng X, Chen Z, Zhang F, Wang N., Studies using concentric ring bifocal and peripheral add multifocal contact lenses to slow myopia progression in school-aged children: a meta-analysis. Ophthalmic Physiol Opt. 2017 |  |
| 59 | 10.1097/IAE.0000000000001666 | Hwang CK, Kolomeyer AM, Brucker AJ, Morgan JIW, Nichols CW, Aleman TS., Localized Bilateral Juxtafoveal Photoreceptor Loss in POEMS: A New Association. Retina. 2017 |  |
| 60 | 10.1159/000473701 | Daien V, Le Pape A, Heve D, Villain M, Bremond Gignac D; Collaborators of the Epidemiology and Safety Program (EPISAFE).., Incidence and Characteristics of Congenital Cataract Surgery in France from 2010 to 2012: The EPISAFE Program. Ophthalmic Res. 2017 |  |
| 61 | 10.2337/db16-1364 | Simó R, Hernández C., GLP-1R as a Target for the Treatment of Diabetic Retinopathy: Friend or Foe? Diabetes. 2017 |  |
| 62 | 10.1016/j.ajo.2016.11.009 | Ares WJ, Tonetti D, Yu JY, Monaco EA, Flickinger JC, Lunsford LD., Gamma Knife Radiosurgery for Uveal Metastases: Report of Three Cases and a Review of the Literature. Am J Ophthalmol. 2017 |  |
| 63 | 10.1007/s40618-017-0608-z | Bartalena L, Veronesi G, Krassas GE, Wiersinga WM, Marcocci C, Marinò M, Salvi M, Daumerie C, Bournaud C, Stahl M, Sassi L, Azzolini C, Boboridis KG, Mourits MP, Soeters MR, Baldeschi L, Nardi M, Currò N, Boschi A, Bernard M, von Arx G, Perros P, et al., Does early response to intravenous glucocorticoids predict the final outcome in patients with moderate-to-severe and active Graves' orbitopathy? J Endocrinol Invest. 2017 |  |
| 64 | 10.1016/j.jneuroim.2017.08.003 | Palterer B, Brugnolo F, Sieni E, Barilaro A, Parronchi P., Neuromyelitis optica, atypical hemophagocytic lymphohistiocytosis and heterozygous perforin A91V mutation. J Neuroimmunol. 2017 |  |
| 65 | 10.1111/ped.13149 | Demircioğlu Kılıc B, Akbalık Kara M, Ozcelik AA, Ustunsoy H, Balat A., Brachial artery pseudoaneurysm: Rare finding in childhood Behcet's disease. Pediatr Int. 2017 |  |
| 66 | 10.3233/JAD-161190 | Shellington EM, Heath M, Gill DP, Petrella RJ., Long-Term Maintenance of Executive-Related Oculomotor Improvements in Older Adults with Self-Reported Cognitive Complaints Following a 24-Week Multiple Modality Exercise Program. J Alzheimers Dis. 2017 |  |
| 67 | 10.1055/s-0043-100116 | Li J, Cai Y, Sun X, Yao D, Xia J., MiR-346 and TRAb as Predicative Factors for Relapse in Graves' Disease Within One Year. Horm Metab Res. 2017 |  |
| 68 | 10.1097/ICO.0000000000001100 | Farooq AV., Herpes Simplex Virus Keratitis and Resistance to Acyclovir. Cornea. 2017 |  |
| 69 | 10.1080/02713683.2017.1341534 | Modrzejewska M, Wiącek MP., A Novel Approach to the Differentiation of Intrabulbar Tumors in Color Doppler Imaging. Curr Eye Res. 2017 |  |
| 70 | 10.1080/02713683.2017.1358373 | Yamada Y, Suzuma K, Onizuka N, Uematsu M, Mohamed YH, Kitaoka T., Evaluation of retinal blood flow before and after panretinal photocoagulation using pattern scan laser for diabetic retinopathy. Curr Eye Res. 2017 |  |
| 71 | 10.1097/IOP.0000000000000723 | Chisholm SAM, Couch SM, Custer PL., Etiology and Management of Allergic Eyelid Dermatitis. Ophthal Plast Reconstr Surg. 2017 |  |
| 72 | 10.1016/j.expneurol.2016.08.004 | Eapen BC, Murphy DP, Cifu DX., Neuroprosthetics in amputee and brain injury rehabilitation. Exp Neurol. 2017 |  |
| 73 | 10.1136/bjophthalmol-2016-309460 | Bhardwaj M, Sen S, Chosdol K, Sharma A, Pushker N, Kashyap S, Bakhshi S, Bajaj MS., miRNA-200c and miRNA-141 as potential prognostic biomarkers and regulators of epithelial-mesenchymal transition in eyelid sebaceous gland carcinoma. Br J Ophthalmol. 2017 |  |
| 74 | 10.1136/jnnp-2017-315782 | Evoli A, Alboini PE, Iorio R, Damato V, Bartoccioni E., Pattern of ocular involvement in myasthenia gravis with MuSK antibodies. J Neurol Neurosurg Psychiatry. 2017 |  |
| 75 | 10.3928/01913913-20170309-01 | Rajavi Z, Feizi M, Behradfar N, Yaseri M, Sayanjali S, Motevaseli T, Sabbaghi H, Faghihi M., Inferior Oblique Overaction: Anterior Transposition Versus Myectomy. J Pediatr Ophthalmol Strabismus. 2017 |  |
| 76 | 10.1080/02713683.2016.1223317 | Jia Y, Hu DN, Sun J, Zhou J., Correlations Between MMPs and TIMPs Levels in Aqueous Humor from High Myopia and Cataract Patients. Curr Eye Res. 2017 |  |
| 77 | 10.1097/IOP.0000000000000536 | Cole SC, Eftekhari K, Oberg T, Mamalis N, Anderson RL., Osteolytic Sarcoidosis of the Orbital Roof Masquerading as a Malignant Orbital Lesion. Ophthal Plast Reconstr Surg. 2017 |  |
| 78 | 10.1111/desc.12379 | Hadad BS, Maurer D, Lewis TL., The role of early visual input in the development of contour interpolation: the case of subjective contours. Dev Sci. 2017 |  |
| 79 | 10.3109/13816810.2016.1164192 | Topçu V, Alp MY, Alp CK, Bakır A, Geylan D, Yılmazoğlu MÖ., A new familial case of Jalili syndrome caused by a novel mutation in CNNM4. Ophthalmic Genet. 2017 |  |
| 80 | 10.1016/j.ophtha.2017.07.010 | Pflugfelder SC, de Paiva CS., The Pathophysiology of Dry Eye Disease: What We Know and Future Directions for Research. Ophthalmology. 2017 |  |
| 81 | 10.14670/HH-11-820 | Kimura A, Namekata K, Guo X, Harada C, Harada T., Dock3-NMDA receptor interaction as a target for glaucoma therapy. Histol Histopathol. 2017 |  |
| 82 | 10.1016/j.oftal.2016.03.021 | Cañizares B, Yago I, Piñero Á, Ruiz M., Unilateral persistent fetal vasculature coexisting with anterior segment dysgenesia. Arch Soc Esp Oftalmol. 2017 |  |
| 83 | 10.1136/jmedgenet-2016-104212 | Gerber S, Ding MG, Gérard X, Zwicker K, Zanlonghi X, Rio M, Serre V, Hanein S, Munnich A, Rotig A, Bianchi L, Amati-Bonneau P, Elpeleg O, Kaplan J, Brandt U, Rozet JM., Compound heterozygosity for severe and hypomorphic NDUFS2 mutations cause non-syndromic LHON-like optic neuropathy. J Med Genet. 2017 |  |
| 84 | 10.1093/hmg/ddx168 | Whitman MC, Engle EC., Ocular congenital cranial dysinnervation disorders (CCDDs): insights into axon growth and guidance. Hum Mol Genet. 2017 |  |
| 85 | 10.1016/j.ophtha.2017.01.027 | Wu Z, Saunders LJ, Daga FB, Diniz-Filho A, Medeiros FA., Frequency of Testing to Detect Visual Field Progression Derived Using a Longitudinal Cohort of Glaucoma Patients. Ophthalmology. 2017 |  |
| 86 | 10.1038/eye.2017.34 | Midena E, Pilotto E., Microperimetry in age: related macular degeneration. Eye (Lond). 2017 |  |
| 87 | 10.1097/IOP.0000000000000806 | Hirabayashi KE, Kalin-Hajdu E, Brodie FL, Kersten RC, Russell MS, Vagefi MR., Retrobulbar Injection of Amphotericin B for Orbital Mucormycosis. Ophthal Plast Reconstr Surg. 2017 |  |
| 88 | 10.1097/ICU.0000000000000456 | Sozeri Y, Salim S., Anticlotting agents and the surgical management of glaucoma. Curr Opin Ophthalmol. 2018 |  |
| 89 | 10.1097/IAE.0000000000001368 | Yong Huang X, Wu N, Li N, Wang Y, Shakoor A., Diagnostic and Therapeutic Challenges. Retina. 2017 |  |
| 90 | 10.1007/s12031-016-0869-6 | D'Amico AG, Maugeri G, Bucolo C, Saccone S, Federico C, Cavallaro S, D'Agata V., Nap Interferes with Hypoxia-Inducible Factors and VEGF Expression in Retina of Diabetic Rats. J Mol Neurosci. 2017 |  |
| 91 | 10.1097/ICO.0000000000001129 | Nakagawa H, Hattori T, Koike N, Ehara T, Narimatsu A, Kumakura S, Matsumoto T, Goto H., Number of Bacteria and Time of Coincubation With Bacteria Required for the Development of Acanthamoeba Keratitis. Cornea. 2017 |  |
| 92 | 10.1097/IAE.0000000000001408 | Pertl L, Haas A, Hausberger S, Pichler T, Rabensteiner DF, Seidel G, Malle EM, Weger M., CHANGE OF CHOROIDAL VOLUME IN UNTREATED CENTRAL SEROUS CHORIORETINOPATHY. Retina. 2017 |  |
| 93 | 10.1002/dmrr.2860 | Omori Y, Yanagisawa K, Sato A, Uchigata Y., The importance of nonstop treatment after delivery for pregnant women with type 2 diabetes. Diabetes Metab Res Rev. 2017 |  |
| 94 | 10.1016/j.anai.2017.01.027 | Bork K, Brehler R, Witzke G, Boor S, Heineke W, Hardt J., Blindness, tetraspasticity, and other signs of irreversible brain damage in hereditary angioedema. Ann Allergy Asthma Immunol. 2017 |  |
| 95 | 10.1007/s10067-016-3417-4 | Vitale A, Emmi G, Lopalco G, Gentileschi S, Silvestri E, Fabiani C, Urban ML, Frediani B, Galeazzi M, Iannone F, Rigante D, Cantarini L., Adalimumab effectiveness in Behçet's disease: short and long-term data from a multicenter retrospective observational study. Clin Rheumatol. 2017 |  |
| 96 | 10.1038/nprot.2017.032 | Lückoff A, Scholz R, Sennlaub F, Xu H, Langmann T., Comprehensive analysis of mouse retinal mononuclear phagocytes. Nat Protoc. 2017 |  |
| 97 | 10.1097/ICO.0000000000001038 | Micali A, Roszkowska AM, Postorino EI, Rania L, Aragona E, Wylegala E, Nowinska A, Ieni A, Calimeri S, Pisani A, Aragona P, Puzzolo D., Comparative Confocal and Histopathological Study of Corneal Changes in Multiple Myeloma. Cornea. 2017 |  |
| 98 | 10.1097/ICU.0000000000000385 | Daya SM., Conjunctival-limbal autograft. Curr Opin Ophthalmol. 2017 |  |
| 99 | 10.1007/s10384-016-0490-9 | Shalaby U., Diltiazem co treatment with cyclosporine for induction of disease remission in sight-threatening non-infectious intraocular inflammation. Jpn J Ophthalmol. 2017 |  |
| 100 | 10.1016/j.ajo.2016.12.023 | Tavares Ferreira J, Proença R, Alves M, Dias-Santos A, Santos BO, Cunha JP, Papoila AL, Abegão Pinto L., Retina and Choroid of Diabetic Patients Without Observed Retinal Vascular Changes: A Longitudinal Study. Am J Ophthalmol. 2017 |  |
| 101 | 10.1016/j.jcjo.2016.07.026 | Eom Y, Song JS, Kim HM., Spectacle plane add power of multifocal intraocular lenses according to effective lens position. Can J Ophthalmol. 2017 |  |
| 102 | 10.1097/IAE.0000000000001401 | Tye A, Klufas MA, McCannel CA, McCannel TA., Intracameral Air in Phacovitrectomy for Maintaining Intraocular Lens Position. Retina. 2017 |  |
| 103 | 10.1111/aos.13385 | Jacobsen AG, Brost B, Vorum H, Hargitai J., Functional benefits and patient satisfaction with upper blepharoplasty - evaluated by objective and subjective outcome measures. Acta Ophthalmol. 2017 |  |
| 104 | 10.1136/bjophthalmol-2016-309504 | Himori N, Kunikata H, Kawasaki R, Shiga Y, Omodaka K, Takahashi H, Miyata T, Nakazawa T., The association between skin autofluorescence and mean deviation in patients with open-angle glaucoma. Br J Ophthalmol. 2017 |  |
| 105 | 10.1007/s00417-017-3696-8 | Callizo J, Pfeiffer S, Lahme E, van Oterendorp C, Khattab M, Bemme S, Kulanga M, Hoerauf H, Feltgen N., Risk of progression in macula-on rhegmatogenous retinal detachment. Graefes Arch Clin Exp Ophthalmol. 2017 |  |
| 106 | 10.1111/cup.12909 | Motaparthi K., Pseudoherpetic transient acantholytic dermatosis (Grover disease): case series and review of the literature. J Cutan Pathol. 2017 |  |
| 107 | 10.1097/IOP.0000000000000837 | Charles NC, Jakobiec FA, Patel P., Periorbital Dermoid Cyst With Unique Trichilemmal Differentiation. Ophthal Plast Reconstr Surg. 2017 |  |
| 108 | 10.1080/09286586.2016.1255763 | Sasongko MB, Agni AN, Wardhana FS, Kotha SP, Gupta P, Widayanti TW, Supanji, Widyaputri F, Widyaningrum R, Wong TY, Kawasaki R, Wang JJ, Pawiroranu S., Rationale and Methodology for a Community-Based Study of Diabetic Retinopathy in an Indonesian Population with Type 2 Diabetes Mellitus: The Jogjakarta Eye Diabetic Study in the Community. Ophthalmic Epidemiol. 2017 |  |
| 109 | 10.1111/jnc.14070 | Bordone MP, González Fleitas MF, Pasquini LA, Bosco A, Sande PH, Rosenstein RE, Dorfman D., Involvement of microglia in early axoglial alterations of the optic nerve induced by experimental glaucoma. J Neurochem. 2017 |  |
| 110 | 10.1016/j.ajpath.2017.08.025 | Kumar S, Nakashizuka H, Jones A, Lambert A, Zhao X, Shen M, Parker M, Wang S, Berriochoa Z, Fnu A, VanBeuge S, Chévez-Barrios P, Tso M, Rainier J, Fu Y., Proteolytic Degradation and Inflammation Play Critical Roles in Polypoidal Choroidal Vasculopathy. Am J Pathol. 2017 |  |
| 111 | 10.1017/S0031182017000270 | Chen SJ, Zhang YX, Huang SG, Lu FL., Galectins expressed differently in genetically susceptible C57BL/6 and resistant BALB/c mice during acute ocular Toxoplasma gondii infection. Parasitology. 2017 |  |
| 112 | 10.1111/aos.13226 | Wirth MA, Freiberg F, Pfau M, Wons J, Becker MD, Michels S., Optical coherence tomography angiography in age-related macular degeneration: persistence of vascular network in quiescent choroidal neovascularization. Acta Ophthalmol. 2017 |  |
| 113 | 10.1136/bjophthalmol-2015-307837 | Dhalla K, Cousens S, Murdoch IE., Phacoemulsification compared with phacotrabeculectomy surgery: a within-person observational cohort study. Br J Ophthalmol. 2017 |  |
| 114 | 10.3171/2016.12.SPINE16540 | Ibrahim TF, Sweis RT, Nockels RP., Reversible postoperative blindness caused by bilateral status epilepticus amauroticus following thoracolumbar deformity correction: case report. J Neurosurg Spine. 2017 |  |
| 115 | 10.1097/ICO.0000000000001263 | Oliva-Biénzobas V, Navas A, C Astiazarán M, Chacón-Camacho OF, A Bermúdez-Magner J, Takane M, Graue-Hernández E, Zenteno JC., CYP1B1 Cytopathy: Uncommon Phenotype of a Homozygous CYP1B1 Deletion as Internal Corneal Ulcer of Von Hippel. Cornea. 2017 |  |
